# Regulation of cofilin phosphorylation in glomerular podocytes by testis specific kinase 1 (TESK1)

**DOI:** 10.1038/s41598-018-30115-3

**Published:** 2018-08-16

**Authors:** Liming Wang, Anne F. Buckley, Robert F. Spurney

**Affiliations:** 1 Division of Nephrology, Department of Medicine, Duke University and Durham VA Medical Centers, Durham, NC 27710 USA; 2 Department of Pathology, Duke University Medical Center, Durham, NC 27710 USA

## Abstract

Expression of a constitutively active Rho A (V14Rho) in podocytes *in vivo* induces albuminuria and foot process (FP) effacement. These effects may be mediated by the Rho A effector Rho kinase (ROK); but inhibition of ROK with Y27632 failed to attenuate albuminuria or FP effacement in V14Rho mice. ROK activates LIM kinases (LIMKs), which phosphorylate and inhibit the actin depolymerizing factor cofilin 1 (CFL1). Sustained phosphorylation of CFL1 is implicated in human nephrotic diseases, but Y27632 did not inhibit phosphorylation of CFL1 *in vivo*, despite effective ROK inhibition. CFL1 is also phosphorylated by testis-specific kinase 1 (TESK1) on the same serine residue. TESK1 was expressed in podocytes, and, similar to the *in vivo* situation, Y27632 had little effect on phospho-CFL1 (pCFL1) levels in cultured podocytes. In contrast, Y27632 reduced pCFL1 levels in TESK1 knockout (KO) cells. ROK inhibition enhanced podocyte motility but, the motility promoting effect of Y27632 was absent in TESK1 KO podocytes. Thus, TESK1 regulates podocyte cytoskeletal dynamics in glomerular podocytes and may play an important role in regulating glomerular filtration barrier integrity in glomerular disease processes.

## Introduction

Glomerular podocytes are highly differentiated cells that cover the external surface of the glomerular blood vessels, and maintain the structural and functional integrity of the kidney’s glomerular filter^1^. Podocyte function is regulated by small GTPases belonging to the Rho GTPase family^2–4^. These small GTPases act as molecular switches controlling activation of multiple downstream effector molecules^5–8^. Among their pleiotropic actions, Rho-dependent signaling cascades modulate cellular morphology and actin polymerization, adhesion, cell migration, proliferation and apoptosis as well as participate in contractile responses^5–8^. While these actions serve homeostatic functions under normal physiologic conditions, Rho-dependent signaling cascades are dysregulated in glomerular disease processes^9–17^. Rho A also has an important homeostatic function by promoting a podocyte phenotype that inhibits cellular motility and stabilizes the glomerular architecture^2–4^. To study the role for Rho GTPases in glomerular homeostasis and pathological processes, we created transgenic (TG) mice that expressed a constitutively active Rho A (V14Rho) specifically in podocytes using a doxycycline inducible strategy^18^. In these transgenic (TG) mice, induction of V14Rho in podocytes caused albuminuria and FP effacement^18^.

Numerous studies suggest that inhibition of the Rho A effector Rho kinase (ROK) has beneficial effects in glomerular disease processes^9–17^. We, therefore, investigated the effect of the ROK inhibitor Y27632^19^ in TG mice expressing V14Rho specifically in podocytes^18^. Unexpectedly, we found that treatment with Y27632 did not reduce albuminuria or FP effacement in V14Rho mice. The inability of Y27632 to reduce albuminuria did not appear to result from an ineffective dosage of Y27632, but was associated with sustained phosphorylation of the actin-depolymerizing factor CFL1 on serine 3, a downstream target of ROK signaling^20^. This may have implications for proteinuric kidney diseases because pCFL1 inhibits its actin-depolymerizing activity^20^, and CFL1 deficiency promotes proteinuria in animal models^21^. Moreover, pCFL1 is absent in glomerular podocytes in normal human kidney tissue, but is enhanced in human glomerular disease processes^22^.

CFL1 is also phosphorylated on the same serine residue (serine 3) by TESK1^20,23^. While the tissue distribution of TESK1 is restricted^20^, a previous study suggested TESK1 may be expressed in podocytes^24^. In support of this observation, we found that TESK1 was expressed in both mouse and human podocytes *in vitro*, and in human kidney biopsy specimens. We, therefore, examined the role of TESK1 in regulating CFL1 phosphorylation and cytoskeletal dynamics in cultured podocytes. Our findings suggest an important role for TESK1 in regulating the podocyte actin cytoskeleton.

## Results

### ROK inhibition does not attenuate V14Rho induced albuminuria

In previous studies, we created TG mice that expressed the constitutively active RhoA transgene (V14Rho mice) specifically in podocytes in a doxycycline inducible fashion^18^. For the studies, 2 TG animals are required to create mice that express V14Rho in podocytes. The first TG animal expresses the reverse tetracycline-controlled transcriptional activator (rtTA) under the control of the human podocin (NPHS2) promoter^25^. The second TG mouse expresses V14Rho under the control of tet operator sequence (tetO) and a minimal CMV promoter (PminCMV)^18^. By breeding the two TG mice, animals are obtained that express both transgenes. In these “double” TG mice (termed V14 Rho mice), treatment with doxycycline induces transgene expression. Controls include “single” TG animals and non-TG mice, which do not express the transgene following doxycycline treatment.

**Figure 1:**
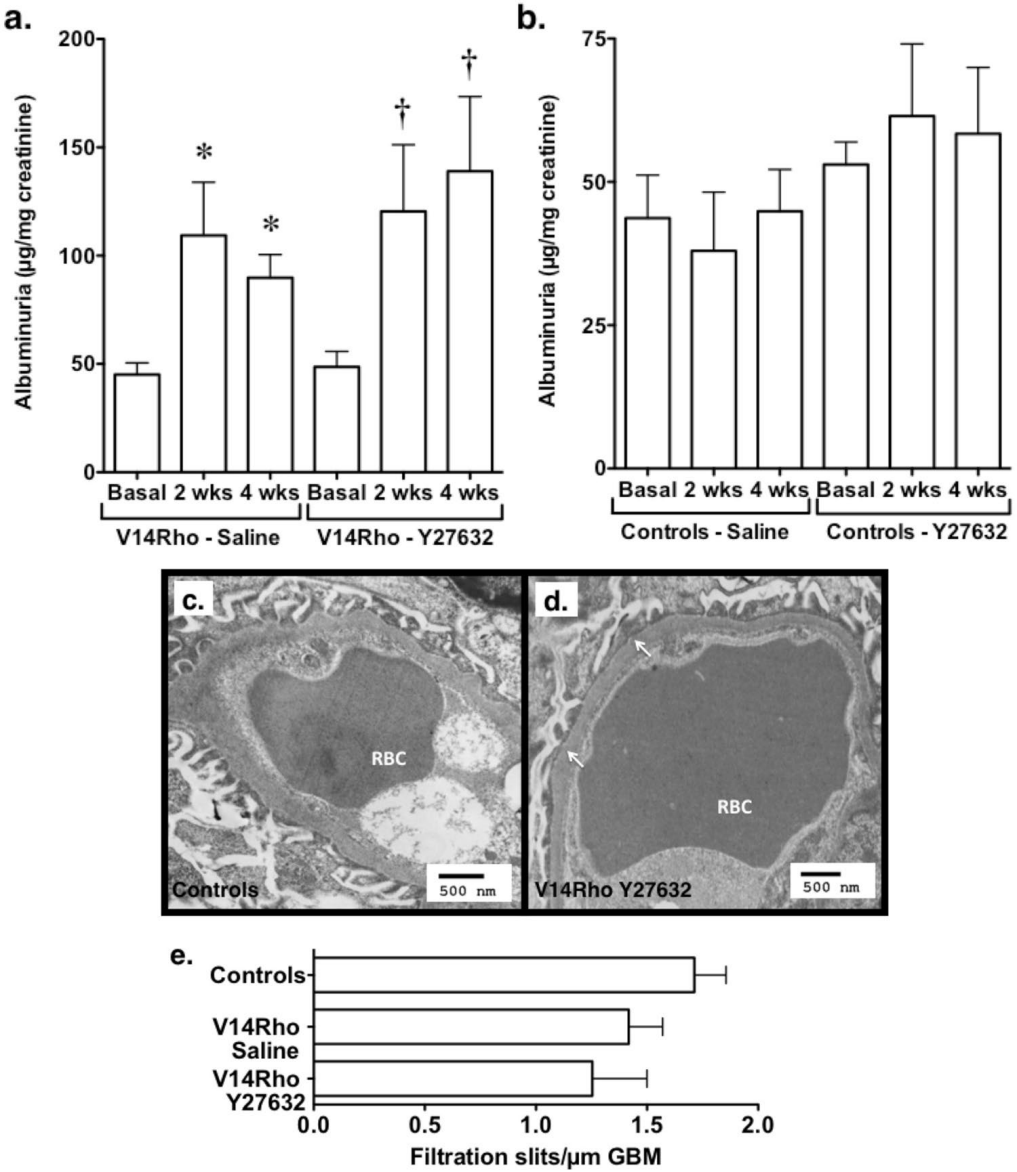
Effect of ROK inhibition on Rho A induced albuminuria and FP effacement. (**a**,**b**) V14Rho (panel a) and control mice (do not express V14Rho) (panel b) were treated with doxycycline for 2 weeks followed by doxycycline and either Y27632 or saline vehicle for an additional 2 weeks. Albuminuria was significantly increased in V14Rho mice compared to baseline at the 2-week and 4-week time points in both vehicle- and Y27632-treated V14Rho mice. In contrast, no significant changes in albuminuria were detected in control mice, which do not express the transgene following doxycycline treatment. (**c**,**d**) Representative pictures of glomerular ultrastructure are shown for both controls and V14Rho mice treated with Y27632. Arrows indicate focal areas of FP flattening and effacement. (**e**) The number of filtration slits per µm of GMB tended to be reduced in V14Rho mice treated with either saline or Y27632. For the albuminuria studies, 7–31 mice were studied in each group. For the ultrastructural experiments, 2 mice were studied in each group. Red blood cells (RBCs) are also labeled in the capillary loops. *P < 0.05 vs basal (saline treated group), ^†^P < 0.05 vs basal (Y27632 treated group).

In previous studies, we found that expression of the V14Rho transgene induced albuminuria^18^. To determine if ROK inhibition attenuated this albuminuric response, we treated V14Rho mice with the ROK inhibitor Y27632^19^. For the studies, V14Rho mice and controls were treated with doxycycline for 2 weeks followed by doxycycline and either Y27632 or saline vehicle for an additional 2 weeks. Urine collections were performed at baseline and at the 2-week and 4-week time points. As shown in Fig. 1a, albuminuria was significantly increased compared to baseline at the 2-week time point. At the 4-week time point, albuminuria was similar to the 2-week time point in both groups and remained elevated compared to baseline. Controls were treated in a similar fashion with doxycycline and either Y27532 or saline vehicle without inducing a significant change in albuminuria (Fig. 1b). Systemic blood pressure (BP) was not affected by treatment with Y27632 (Table 1).

**Table 1:** Effect of Y27632 on systolic blood pressure.

Treatment	Baseline	End of Treatment
Saline control	133 ± 2.3	137 ± 5.0
Y27632	136 ± 3.1	136 ± 2.2

Blood pressure was measured in 10 mice in each group.

In our previous study^18^, light microscopic findings were unremarkable in the V14Rho mice but FP effacement was observed by transmission electron microscopy (TEM). We, therefore, investigated the effect of Y27632 in controls and V14Rho mice treated with Y27632. As shown in Fig. 1c, controls showed only mild or no FP effacement. In the V14Rho mice, patchy FP effacement was observed, consistent with previous studies^18^, which was more prominent in the V14Rho mice treated with Y27632 (Fig. 1d). Thus, there was no qualitative improvement in FP effacement with Y27632 treatment. Consistent with these observations, the number of slit diaphragms/µm glomerular basement membrane (GBM) tended to be reduced in both groups of V14Rho mice compared to controls (Fig. 1e).

### Y27632 does not inhibit V14Rho induced phosphorylation of CFL1

**Figure 2:**
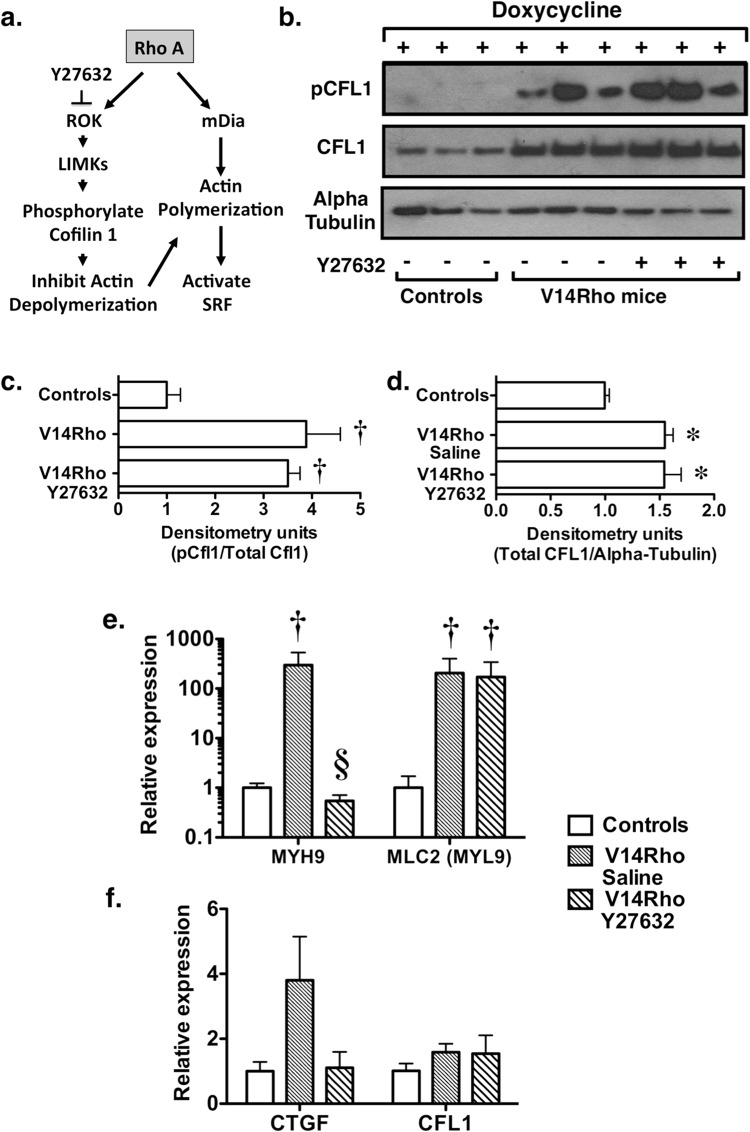
Effect of Rho A on CFL1 phosphorylation *in vivo*. (**a**) Rho A activates ROK which stimulates LIMKs to phosphorylate CFL1 and inhibit its depolymerizing activity. Rho A also activates mDia, which promotes actin polymerization and activates SRF (serum response factor). SRF has potent effects on induction of structural and regulatory elements of the actin cytoskeleton including CFL1 (see text). (**b–d**) Induction of V14Rho by doxycycline enhanced phosphorylation of CFL1, which was associated with a robust increase in CFL1 protein levels. Treatment with Y27632 did not affect the phosphorylation state of CFL1. (**e**,**f**) Expression of V14Rho in podocytes induced expression of MYH9 (myosin heavy chain 9), MLC2 (also termed myosin light chain 2), CTGF (connective tissue growth factor) and CFL1 (cofilin-1). After correcting for multiple comparisons, these differences were significant for MYL9 and MYH9. For the immunoblotting experiments, 6 mice were studied per experimental group. For the quantitative RT-PCR experiments 7–9 mice were studied per experimental group. *P < 0.05 or ^†^P < 0.01 vs controls (do not express the V14Rho transgene following doxycycline treatment), ^§^P < 0.01 vs V14Rho treated with saline.

An important downstream target of ROK is the actin-depolymerizing factor CFL1. As shown in Fig. 2a, ROK activates LIM (Lin11, Isl-1 & Mec-3 domain) kinases (LIMKs), which phosphorylate and inhibit the depolymerizing activity of CFL1^20^. Rho A also activates mDia (mammalian homolog of Diaphanous) and promotes actin polymerization^26^. This increase in actin polymerization stimulates serum response factor (SRF), a transcription factor that regulates expression of multiple structural and regulatory elements of the actin cytoskeleton^27,28^. ROK-dependent CFL1 inhibition cooperates with mDia to promote polymerization of the actin cytoskeleton and stimulate serum response factor^29^. Figure 2b,c shows the effects of V14Rho induction and Y27632 on pCFL1 levels in enriched glomerular preparations. Unexpectedly, Y27632 had little effect on CFL1 phosphorylation. The alterations in pCFL1 levels were accompanied by large changes in the protein levels of total CFL1 (Fig. 2b–d). These changes in CFL1 protein levels likely resulted from Rho A dependent activation of SRF, which induces CFL1^28^. Figure 2e,f shows the effects of V14Rho induction and Y27632 treatment on expression of mRNAs of other SRF responsive genes in the enriched glomerular preparations. Induction of V14Rho caused a significant increase in MYL9 and MYH9.

### Y27632 effectively inhibited ROK activity *in vivo*

**Figure 3:**
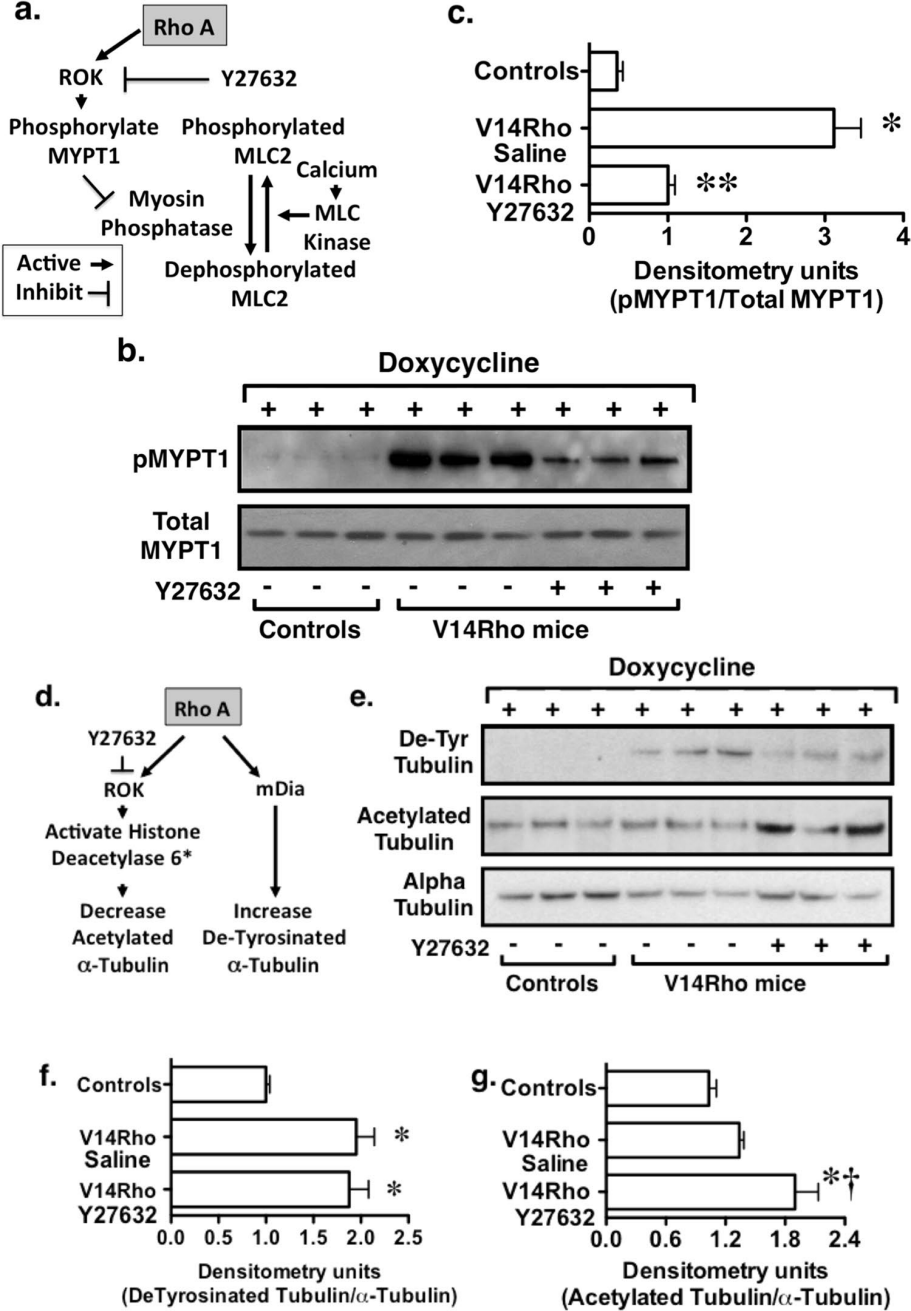
Effects of ROK inhibition *in vivo*. (**a**) Myosin light chain 2 (MLC2) is phosphorylated by MLC kinase by calcium dependent mechanisms. Rho A activates ROK and inhibits myosin phosphatase by phosphorylating MYPT1 (pMYPT1) which, in turn, inhibits dephosphorylation MLC2. (**b**,**c**) Induction of V14Rho with doxycycline enhanced phosphorylation of MYPT1 and this effect was reduced by Y27632. (**d**) Activation of ROK by Rho A stimulates histone deacetylase 6 and decreases acetylated α-tubulin. Rho A also actives mDia (mammalian homolog of Diaphanous) independently of ROK and promotes formation of detyrosinated α-tubulin (D-Tyr tubulin). (**e**–**g**) Induction of V14Rho in podocytes promoted formation of detyrosinated α-tubulin in enriched glomerular preparations and Y27632 had little effect on V14Rho induced detyrosinated α-tubulin levels. Acetylated α-tubulin was observed at baseline and was not changed by induction of V14Rho, perhaps due to the high basal levels of acetylated α-tubulin in preparation containing a mixed population of cells. In contrast, Y27632 enhanced acetylated α-tubulin levels. For the experiments, 6 mice were studied per experimental group. *P < 0.01 vs controls (do not express the V14Rho transgene following doxycycline treatment), **P < 0.01 vs V14Rho treated with saline, ^†^P < 0.05 vs V14Rho treated with saline.

We next examined the effectiveness of ROK inhibition *in vivo*. As shown in Fig. 3a, myosin light chain 2 (MLC2) is phosphorylated by MLC kinase in a calcium dependent fashion^30^. ROK inhibits myosin phosphatase and increases actomyosin contractility by phosphorylating MYPT1 (myosin phosphatase targeting/regulatory subunit 1) and, in turn, inhibiting dephosphorylation of MLC2 by myosin phosphatase^30^. To investigate the effectiveness of ROK blockade *in vivo*, we measured the effect Y27632 on phosphorylation of MYPT1. As shown in Fig. 3b,c, MYPT1 phosphorylation was induced by doxycycline in V14Rho mice compared to saline treated controls, and this increase in MYPT1 phosphorylation was inhibited by Y27632, suggesting effective ROK inhibition *in vivo*.

According to current models, Rho A dependent ROK activation also stimulates histone deacetylase 6 and decreases acetylated α-tubulin^31^. As a result, Y27632 inhibits histone deacetylase 6 and increases acetylated α-tubulin. In contrast, Rho A activates mDia independent of ROK activation, and promotes formation of detyrosinated α-tubulin as shown in Fig. 3d^26^. To investigate the effect of V14Rho induction and Y27632 on the microtubule cytoskeleton, we measured detyrosinated and acetylated α-tubulin levels in enriched glomerular preparations. As shown in Fig. 3e–g, induction of V14Rho promoted formation of detyrosinated α-tubulin and Y27632 had little effect on V14Rho induced detyrosinated α-tubulin levels consistent with a ROK independent effect. Acetylated α-tubulin was detected at baseline and we did not detect a decrease in acetylated α-tubulin levels in podocytes after induction of V14Rho, perhaps due to differences in the high levels of acetylated α-tubulin in other cells types in the glomerular preparations containing a mixed population of cells. Y27632, however, enhanced acetylated α-tubulin levels consistent with effective ROK inhibition *in vivo*.

### Expression of TESK1 by glomerular podocytes

**Figure 4:**
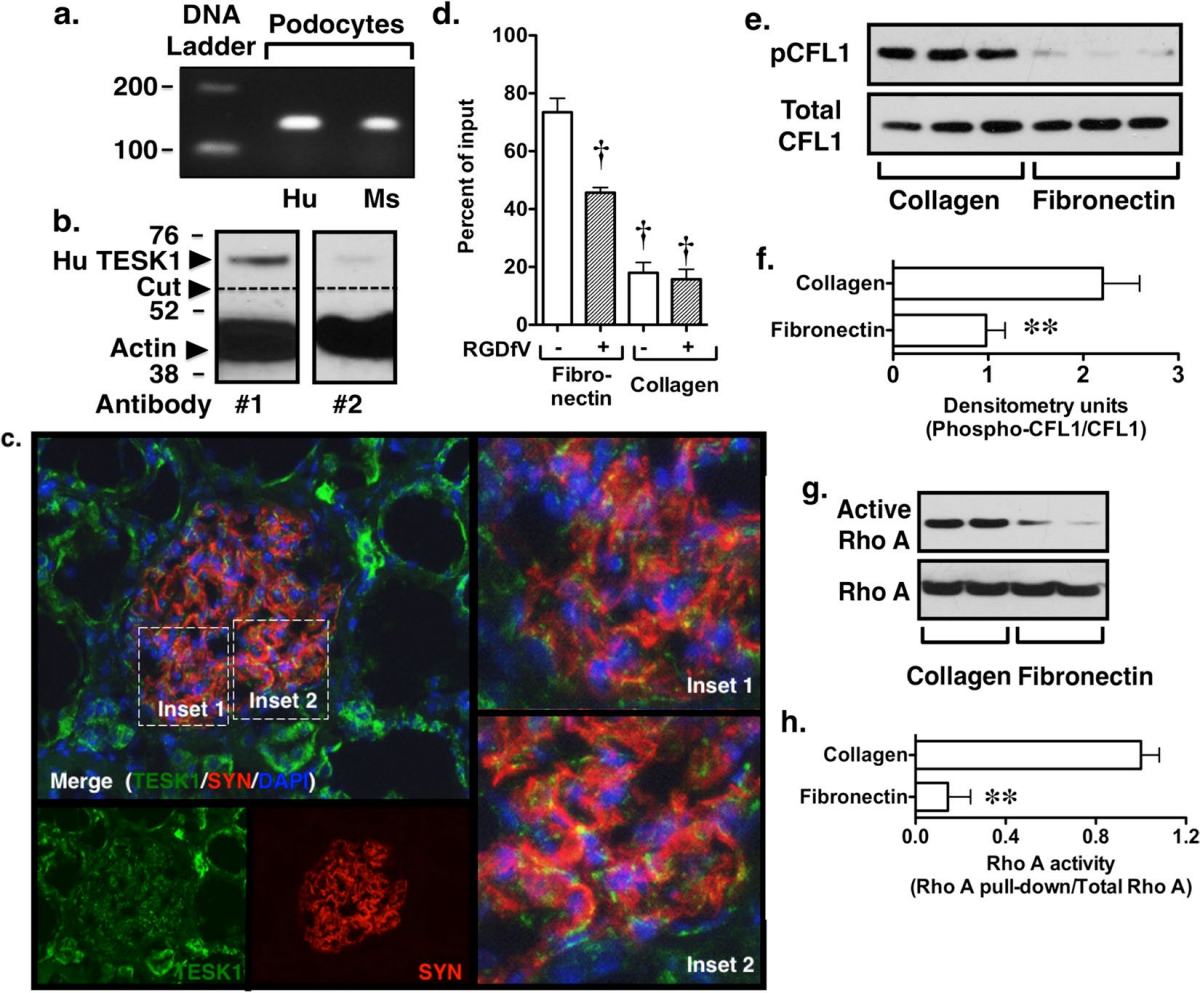
Expression of TESK1 in glomerular podocytes. (**a**) PCR products of the appropriate size were amplified from human (Hu) and mouse (Ms) podocytes using intron spanning primers. DNA sequencing confirmed amplification of TESK1. (Supplementary Figure S1) (**b**) A monoclonal antibody to Hu TESK1 (#1) and polyclonal antibody to TESK1 (#2) confirmed expression of TESK1 in Hu podocytes. The antibody did not react with Ms TESK1. (**c**) We next investigated TESK1 expression in Hu kidney sections using the monoclonal antibody to TESK1 (green) and the podocyte marker synaptopodin (SYN) (red) as described in the Methods Section. Nuclei were counterstained with DAPI (blue). Merging of the images shows that TESK1 co-localized with the podocyte marker SYN at focal subcellular locations within the cell (insets). TESK1 was also found in tubular cells. TESK1 and SYN staining were not detected in the absence of the primary antibodies. (**d**) To determine if podocytes bound fibronectin, we performed attachment assays using fibronectin and collagen coated tissue dishes in the presence or absence of the integrin β3 inhibitor peptide cyclo-RGDfV. Mouse podocyte bound avidly to fibronectin and the binding was inhibited by cyclo-RGDfV. Podocytes bound collagen less avidly and cylco-RGDfV did not significantly affect binding to collagen-coated dishes. (**e**,**f**) Plating podocytes on fibronectin decreased CFL1 phosphorylation compared to cells plated on collagen. (**g**,**h**) Rho A activity was enhanced in podocytes plated on collagen compared to cells plated on fibronectin. **P < 0.01 vs collagen ^†^P < 0.01 fibronectin treated with saline vehicle.

ROK activates LIMKs, which phosphorylate CFL1 on serine 3^20^. TESK1 also phosphorylates CFL1 on this same serine residue^20,23^. While the tissue specific expression of TESK1 is restricted^20^, TESK1 is reportedly expressed in proximal renal tubular cells and glomerular podocytes^24^. We used intron-spanning primers to screen for TESK1 mRNA expression in cultured podocytes^32^. As shown in Fig. 4a, a PCR product of the appropriate size was detected in mouse podocytes, and sequencing of the PCR product confirmed amplification of the mouse TESK1 sequence^32^ (Supplementary Figure S1). Using the analogous human primer pairs, a RT-PCR product of the appropriate size was also detected in a human podocyte cell line. As shown in Fig. 4b, we were also able to detect immunoreactive TESK1 in human podocyte lysates using either a monoclonal antibody that detects human TESK1 (antibody #1) or a second polyclonal antibody to human TESK1 (antibody #2). Unfortunately, these antibodies did not react with mouse TESK1. We, therefore, investigated TESK1 expression in frozen human kidney tissue sections. Immunofluorescence (IF) studies were performed using antibodies to the podocyte marker synaptopodin (SYN) (rhodamine red-x) and a TESK1 antibody (Alexa Flour 488). Nuclei were counterstained with DAPI. As shown in Fig. 4c, TESK1 co-localized with the podocyte marker synaptopodin at focal subcellular locations within the cell (insets). TESK1 was also found in renal tubular cells as previously described^24^. (See Supplementary Figure S2 for the negative controls for the IF studies.) Taken together, these data provide strong evidence that TESK1 is expressed in mouse and human glomerular podocytes.

### Role of TESK1 in CFL1 phosphorylation

Activation of TESK1 has been linked to cellular adhesion and integrin activation following binding to fibronectin^20,23^. The major integrins expressed by podocytes are integrin αvβ3 and α3β1, which bind fibronectin and laminins, respectively^33,34^. We, therefore, tested the ability of podocytes to attach to either fibronectin or collagen in the presence or absence of the integrin β3 inhibitor peptide cyclo-RGDfV^35^. As shown in Fig. 4d, a significant percentage of the podocytes attached to fibronectin and attachment was inhibited by cyclo-RGDfV. Attachment of podocyte to collagen was reduced compared to fibronectin and was not inhibited by the cyclo-RGDfV peptide.

We next investigated pCFL1 levels in podocytes plated on fibronectin and collagen in the presence and absence of serum. As shown in Fig. 4e,f, binding of podocytes to fibronectin significantly inhibited pCFL1 levels and this effect was associated with a significant decrease in Rho A activity (Fig. 4g,h). These findings are similar to other cell lines expressing aαvβ3 integrins^36–38^. In contrast, plating podocytes on fibronectin in the presence of serum resulted in a significant increase in pCFL1 levels compared to podocytes plated on collagen (Supplementary Figure S3). In support of this observation, serum enhances Rho A activity in αvβ3 integrin expressing NIH3T3 cells^39^ plated on fibronectin, which would tend to promote LIM kinase induced CFL1 phosphorylation and inhibition of CFL1 activity. However, growth factors present in serum have complex effects on Rho GTPases signaling and CFL1 activity. For example, epidermal growth factor (EGF) increases pCFL1 levels in a MTLn3 cells, but EGF induced phosphorylation of cofilin did not inhibit cofilin severing activity in this cell line^40^. Given the complex effects of serum on Rho GTPases and CFL1, we focused our remaining studies on CFL1 phosphorylation in the absence of serum stimulation.

### Effect on TESK1 KO on CFL1 phosphorylation

**Figure 5:**
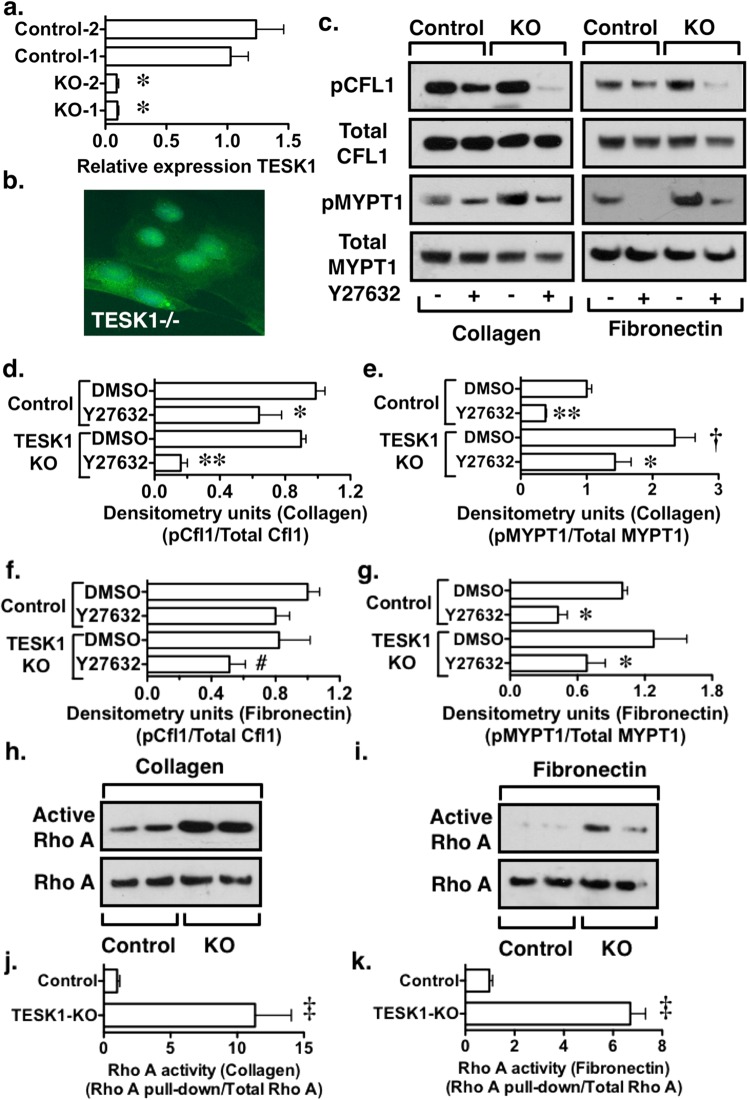
Both ROK and TESK1 play a role in CFL1 phosphorylation. (**a**) Relative expression of TESK1 mRNA was difficult to detect in the clones chosen for study compared to controls. (**b**) Knockout (KO) of TESK1 in mouse (Ms) podocytes resulted in expression of GFP in TESK1 KO cells (see text). Nuclei were counterstained with DAPI. (**c–g**) Y27632 had little affect on pCFL1 levels in control podocytes plated on either collagen or fibronectin, similar to the *in vivo* situation. This result did not result from ineffective ROK inhibition because pMYPT1 levels were reduced by treatment with Y27632 (10 µM). KO of TESK1 also had little effect on pCFL1 levels but tended to enhance MYPT1 phosphorylation. The increase in pMYPT1 levels was significant in podocytes plated on collagen. In contrast to control podocytes, Y27632 (10 µM) potently reduced pCFL1 levels in TESK1 KO cells, consistent with a role for both ROK and TESK1 in promoting phosphorylation of CFL1. (**h**–**k**) TESK1 KO increased Rho A activity in podocytes plated on either collagen or fibronectin. Results of 4 independent experiments. Images were obtained at a magnification of 400x. *P < 0.05 vs either control podocytes (cells transfected with a scrambled construct) or TESK1 KO podocytes treated with DMSO, **P < 0.01 vs either control podocytes or TESK1 KO podocytes treated with DMSO, ^†^P < 0.05 vs KO DMSO ^#^P < 0.05 vs control podocytes treated with DMSO, ^‡^P < 0.001 vs control podocytes.

To further investigate the role of TESK1 in podocyte biology, we knocked out TESK1 in mouse podocytes using CRISPR [(Clustered regularly interspaced short palindromic repeats)/CAS9 (CRISPR associated protein 9)] technology^41^. The strategy chosen utilizes homologous recombination to express green fluorescent protein (GFP) under the regulation of the endogenous TESK1 promoter (see Methods Sections). After selecting with puromycin, 2 clones were chosen for study that did not express TESK1 mRNA by quantitative RT-PCR using the intron-spanning primers described above, which amplify the central portion of the kinase domain of TESK1 (Fig. 5a). As shown in Fig. 5b, both clones were weakly GFP positive. Unfortunately, we could not locate an antibody that reacted with mouse TESK1 to further confirm KO of TESK1 at the protein level. Control cells were transfected with a scrambled construct and were also selected with puromycin (designated controls or control podocytes). For the experiments, control podocytes and KO cells were plated on collagen or fibronectin in the absence of serum. As shown in Fig. 5c–g, Y27632 had a modest effect on pCFL1 levels in control podocytes plated on either collagen or fibronectin (similar to the *in vivo* situation). Moreover, pCFL1 levels were not inhibited by the specific Cdc42 inhibitor ML141^42^ or the specific Rac1 inhibitor NSC237667^43^ (Supplementary Figure S4). The inability of Y27632 to significantly reduce CFL1 phosphorylation did not appear to be due to inadequate ROK inhibition because Y27632 reduced phospho-MYPT1 (pMYPT1) levels in control cells in both groups. Moreover, podocytes expressed the downstream ROK signaling target LIMK (Supplementary Figure S5), suggesting that the Rho-ROK-LIMK pathway was intact. Surprisingly, KO of TESK1 had little effect on baseline CFL1 phosphorylation, but significantly enhanced phosphorylation of MYPT1 in podocytes plated on collagen. In contrast to control podocytes, the combination of TESK1 KO and ROK inhibition potently inhibited CFL1 phosphorylation in TESK1 KO podocytes plated on either collagen or fibronectin. To determine if sustained CFP1 phosphorylation and the increase in pMYPT1 levels in KO cells was due to a change in Rho A activation, we measured Rho A activity using a pull-down assay. As shown in Fig. 5h–k, TESK1 KO cells enhanced Rho A activity in podocytes plated on either collagen or fibronectin. Lastly, the effects of TESK1 KO on pCFL1 and pMYPT1 levels were similar in podocytes plated on fibronectin in the presence of serum (Supplementary Figure S6). These data suggest that both TESK1 and ROK play important roles in regulating CFL1 phosphorylation, which may directly affect its depolymerizing activity.

### Effect of TESK1 KO on the podocyte actin cytoskeleton and podocyte motility

**Figure 6:**
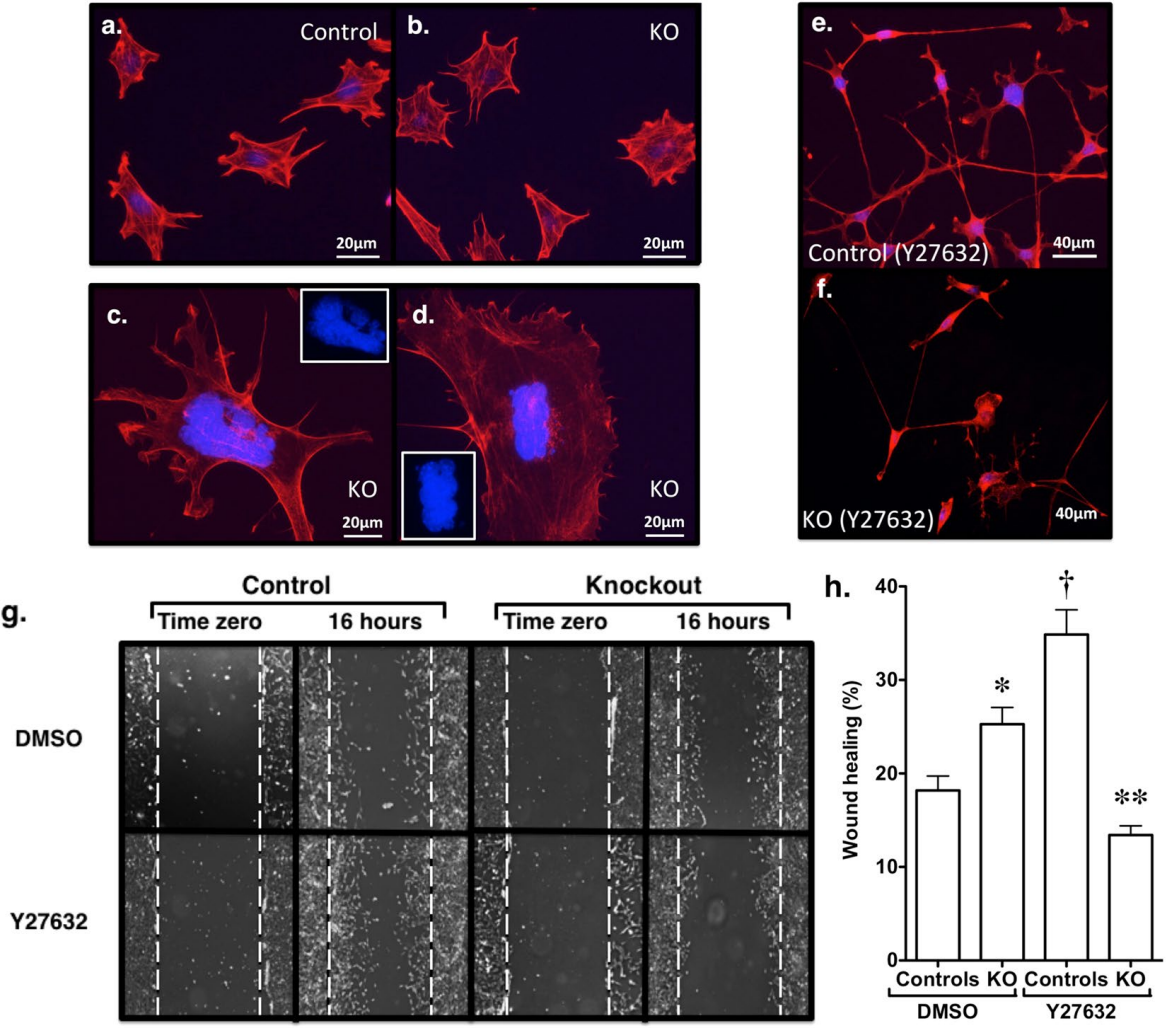
Effect of TESK1 KO on polymerized actin (F-actin) levels. (**a,b**) Representative images of F-actin staining are shown for control and KO podocytes using Alexa Fluor 568 phalloidin in control podocytes and TESK1 KO podocytes. Nuclei were counterstained with DAPI. (**c,d**) A few large podocytes had dysmorphic nuclei resembling multinucleate cells were observed in TESK1 KO podocytes. (**e,f**) Treatment with Y27632 (10 µM) induced an arborized morphology with long cytoplasmic processes in both control and KO podocytes. (**g,h**) Representative image of wound healing assays by treatment group. TESK1 KO significantly enhanced wound healing compared to control podocytes treated with DMSO. ROK inhibition with Y27632 (10 µM) increased wound healing in control podocytes but inhibited wound healing in TESK1 KO podocytes. *P < 0.025 vs control podocytes treated with DMSO, ^†^P < 0.01 vs control podocytes treated with DMSO, **P < 0.05 vs KO podocytes treated with DMSO or control podocytes treated with Y27632.

We next investigated the effect of TESK1 KO on the actin cytoskeleton by staining polymerized actin (F-actin) with Alexa Fluor 568 phalloidin. Nuclei were counterstained with DAPI (4’,6-diamidino-2-phenylindole). As shown in Fig. 6a,b, there were no qualitative differences in morphology of phalloidin stained KO cells compared to control cells treated with vehicle. There were, however, a few large TESK1 KO cells with dysmorphic, angulated nuclear profiles (Fig. 6c,d). In contrast to vehicle treated cells, treatment with Y27632 induced an arborized cellular morphology with long cytoplasmic processes in both control and KO podocytes (Fig. 6e,f), as has been reported by other investigators in podocytes treated with Y27632^44^.

Podocyte motility has been used to study pathways that modulate cytoskeletal reorganization. Indeed, accumulating evidence suggests that alterations in podocyte motility *in vitro* are associated with FP effacement and loss of glomerular filtration barrier integrity *in vivo*^45–49^. To determine if Y27632 induced a motile podocyte phenotype, we used a wound-healing assay as previously described^50^. For the experiments, a wound was created as described in the Methods Section, and then control and TESK1 KO podocytes were incubated overnight (16 hours) with either Y27632 or vehicle. Figure 6g shows representative photographs of the wounds at time zero and at the 16-hour time point. As shown in Fig. 6h, both TESK1 KO and treatment of control cells with Y27632 increased wound healing. In contrast, treatment with Y27632 significantly inhibited wound healing in the TESK1 KO cells. These data suggest that TESK1 plays a role in modulating actin cytoskeletal dynamics.

## Discussion

In the present studies, we found that albuminuria and FP effacement in V14Rho mice were not affected by treatment with the ROK inhibitor Y27632. This effect did not appear to result from an inadequate dosage of Y27632 but was associated with sustained phosphorylation of CFL1, as observed in human glomerular diseases^22^. Because CFL1 is phosphorylated by TESK1^23^ on the same serine residue as ROK-LIMK signaling, we determined if TESK1 contributed to CFL1 regulation in podocytes. We found that: (1) TESK1 was expressed in both mouse and human podocytes, (2) TESK1 expression co-localized with the podocyte marker synaptopodin in human kidney, (3) CFL1 phosphorylation was modestly affected by ROK inhibition in cultured podocytes, similar to the *in vivo* situation, (4) Combined ROK inhibition and TESK1 KO revealed a role for TESK1 in CFL1 phosphorylation, and (5) TESK1 KO promoted podocyte motility but inhibited the motile podocyte phenotype induced by treatment with Y27632. Taken together, these data suggest that TESK1 plays an important role in modulating CFL1 phosphorylation and cytoskeletal dynamics in podocytes.

TESK1 is a protein kinase that has dual specificity for serine/threonine and tyrosine residues^51^. Like ROK-LIMK signaling, TESK1 phosphorylates CFL1 on serine 3 and inhibits its actin depolymerizing activity^20,23^. The mechanisms of TESK1 activation are not well characterized but its kinase activity toward CFL1 is thought to be activated by integrins and cellular adhesion^20,23^. CFL1 is also required for RNA polymerase II-mediated transcription by interacting with actin^20^. Regulation of CFL1 activity plays an important role in glomerular diseases because podocyte specific KO of CFL1 causes sustained proteinuria by 3 months of age and FP effacement by 8 months of age^21^. Moreover, phosphorylation of CFL1 in podocytes is enhanced in human glomerular diseases and is localized to the nucleus^20,22^. In contrast, podocyte CFL1 is not phosphorylated and localizes to the cytoplasm in normal kidney tissue^22^. Taken together these data suggest that pathways that regulate CFL1 activity are likely to play important roles in glomerular disease processes.

The role of the podocyte in maintaining glomerular filtration barrier integrity is thought to require rapid alterations in cytoskeletal dynamics to adapt to the local changes in glomerular hemodynamics^52^. Rapid changes in cytoskeletal reorganization are also required for cellular motility and, therefore, podocyte motility assays have been used to study the cellular pathways that regulate the podocyte cytoskeleton and, in turn, maintain the integrity of the glomerular filtration barrier^52^. Indeed, alterations in podocyte motility *in vitro* are associated with albuminuria and FP effacement *in vivo*^45–49^. Motility requires coordinated activation and inhibition of the signaling pathways that regulate cytoskeletal dynamics^53,54^, including pathways that modulate either assembly or disassembly of actin filaments such as CFL1^55^. Consistent with a coordinated response to TESK1 KO, Rho A activity was potently stimulated in podocytes lacking TESK1. This increase in Rho-ROK-LIMK signaling in KO podocytes maintained CFL1 in a phosphorylated state because combined TESK1 KO and ROK inhibition significantly decreased pCFL1 levels. Thus, TESK1 and Rho-ROK-LIMK signaling may cooperate to modulate CFL1 phosphorylation and activity. Based on these observations, we speculate that the loss of either TESK1 or inhibition of ROK causes the reciprocal, coordinated activation of the other pathway and a modest increase in motility. In contrast, compensation is lost by combined ROK inhibition and TESK1 KO, resulting in poorly coordinated activity of CFL1 and TESK1, and reduced podocyte motility. Additional studies will be necessary to further investigate this possibility.

In addition to cellular motility, cell division also requires coordinated activation and inhibition of pathways that regulate assembly and disassembly of the actin cytoskeleton. Indeed, KO of CFL1 results in aberrant cytokinesis and increases both cell size and the percentage of multinucleated cells^56^. Consistent with these observations, we found that KO of TESK1 resulted in a few large cells with dysmorphic nuclei that be related to aberrant nuclear division. Because podocytes have a limited potential for proliferation *in vivo*^57^, these findings are likely not relevant to most glomerular diseases, but suggest that TESK1 may play a role in cell division during glomerular development. In addition, TESK1 is overexpressed in some cancers such as the renal malignancy Wilms tumor^58^, consistent with a role for TESK1 in cell proliferation.

The importance of ROK in glomerular diseases is predominantly based on pharmacological studies using chemically distinct ROK inhibitors^9–17^. In these studies, ROK inhibition attenuated renal damage in a variety of experimental models^9–17^ and, in most studies, was started before or soon after induction of kidney injury. In the few studies that treatment was delayed, the effects of ROK inhibition on proteinuria were modest^59^ or absent^16^, similar to the findings in the present study. Thus, it may be necessary to initiate ROK inhibition early in the course of disease to be effective. Alternatively, the dosage of Y27632 utilized in the present study may not have been sufficient to affect albuminuria. We think this is unlikely given beneficial effects of Y27632 in animal studies using lower or similar dosages of Y27632^60–62^. Moreover, the dosage of Y27632 utilized in the present study inhibited MYPT1 phosphorylation and enhanced acetylation of α-tubulin *in vivo* suggesting Y27632 effectively blocked the effects of ROK activation *in vivo*. Taken together, we speculate that pharmacologic treatment strategies that target ROKs may require initiating therapy early in the disease process to be an effective treatment strategy.

In summary, we found that glomerular podocytes express that the novel kinase TESK1, which plays a previously unrecognized role in regulating cytoskeletal dynamics in glomerular podocytes. This finding was stimulated by the observation that ROK inhibition did not affect glomerular filtration barrier integrity or FP effacement in mice expressing a constitutively active Rho A construct (V14Rho) specifically in podocytes *in vivo*. This effect did not result from an inability to block ROK activity, but was associated with sustained phosphorylation of CFL1 as observed in human glomerular diseases^22^. Similar to the *in vivo* situation, ROK inhibition had little effect on CFL1 phosphorylation in cultured podocytes. In contrast, ROK inhibition potently reduced pCFL1 levels in TESK1 KO cells. Moreover, TESK1 KO altered podocyte motility in wound healing assays. While further studies will be necessary to determine the specific role of TESK1 in glomerular disease processes *in vivo*, these data suggest an important role for TESK1 in modulating cytoskeletal dynamics in glomerular podocytes, which may be directly relevant to glomerular disease processes.

## Methods

### Materials

The ROCK inhibitor Y27632^63^ was obtained from Tocris Biosciences (Bristol, UK). Collagen was obtained from Sigma-Aldrich, Inc. (product number C8919) and fibronectin obtained from Santa Cruz Biotechnology (product number sc-29011). De-identified frozen human kidney sections were obtained from Origene Technologies (Rockville, MD). The human tissue samples were obtained after providing informed consent using protocols approved by the institutional review board (IRB) in accordance with the Health Insurance Portability and Accountability Act (HIPAA) of the United States of America (https://www.origene.com/products/tissues/tissue-qc).

### Culture of SV40 transformed mouse glomerular epithelial cells

The immortalized mouse podocyte cell line was maintained on collagen coated tissue culture clusters (Corning Inc., Corning, NY) and differentiated as previously described^64^. For the immunoblotting experiments, cells were cultured in serum free medium overnight and then treated with the indicated agents for 1 hour. Cells were solubilized in NP40 lysis buffer (150 mM NaCl, 50 mM hydroxymethyl-aminomethane [Tris] and 1% Nonidet P40) with protease inhibitors (Sigma-Aldrich, product number P8340) and the phosphatase inhibitor calyculin (50 nM) Cell Signaling Technology (produce number 9902) and stored at minus 70 °C until study.

### Conditionally Immortalized Human Podocyte Culture and Reagents

Conditionally immortalized human podocytes were cultured under growth permissive conditions at 33 °C in RPMI 1640 medium (Gibco; Gaithersburg, MD, USA) supplemented with 10% fetal bovine serum (Gibco), penicillin-streptomycin (Invitrogen Life Technologies; Grand Island, NY, USA), and 5% 100x Insulin-Transferrin-Selenium supplement (Invitrogen) as described^50^. Podocyte differentiation was induced by transfer to of the cell cultures to growth restrictive condition at 37 °C.

### Wound healing assays

Podocytes were plated in 6 well tissue culture clusters (Evergreen Scientific, Los Angeles, CA) coated with collagen or fibronectin, and then differentiated for 7–10 days as described^18^. Cell monolayers were then wounded with a 1000 µl pipet tip and incubated for 16 in 10 µM Y27632, 10 µM pyringtegrin (Tocris Bioscience, product number 4987) or DMSO vehicle as previously described^50^. Podocytes were imaged at time zero immediately after applying the wound and 16 hours later using a Nikon Eclipse TE-2000S microscope with a Roper Scientific Photometrics digital camera. The number of pixels in the wound area were measured at time zero, and at the 16-hour time point using Adobe Photoshop CS6 software (Adobe Systems, Inc). Data were expressed as the ratio of the wound area after treatment divided by the wound area at time zero as previously described^50^. Measurements were made without knowledge of treatment group.

### Animals studies

Creation of the both V14Rho construct and V14Rho TG mice have been previously described^18^. To obtain podocyte specific expression or the V14Rho transgene, TG mice were bred with a second TG line in which the podocyte specific human podocin promoter (NPHS2) drives expression of rtTA^25^. Mice expressing both the V14Rho and rtTA genes are obtained from these breeding pairs and in these “double” TG mice treatment with 2-mg/ml doxycycline in drinking water (with 2% sucrose to enhance palatability) induces the V14Rho transgene^18^. Screening for the V14Rho and NPHS2-rtTA transgenes was by PCR as described^18^. All mice were maintained on the FVB/NJ background for the studies.

For the experiments, “double” TG mice were treated with either doxycycline or vehicle (2% sucrose without doxycycline) for 2 weeks to induce the transgene. Previous studies have shown that treatment with 2-mg/ml doxycycline for 1 week maximally induces the transgene^25^. Control mice (“single” TG and non-TG mice) were treated in an identical fashion. Y27632 was administered in saline at a dosage of 10 mg/kg/day by twice daily subcutaneous injections. Twenty-four hour urine collections were obtained at the indicated time points. Mice were then sacrificed and both blood and tissues harvested, and stored at −70 °C for the studies described below. All animal procedures were approved by the Animal Care and Use Committee of Duke and Durham Veterans Administration Medical Centers and all methods were performed in accordance with the relevant guidelines and regulations.

### Urinary albumin excretion

Albuminuria was measured using a kit from AssayPro (St. Charles, MO), urine creatinine levels were measured using a kit from Exocell (Philadelphia, PA) and urinary albumin excretion was expressed as the albumin/creatinine ratio.

### Transmission electron microscopy (TEM)

Small blocks of cortical tissue were fixed in an aqueous solution of 8% glutaraldehyde (Sigma-Aldrich) and TEM samples and images were prepared using standard techniques by the Research Electron Microscopy Service at Duke University, Durham, NC. Analysis at the electron microscopic level was performed in a qualitative fashion and areas of interest chosen in semithin sections for preparation of ultrathin sections for examination by a pathologist (A.F.B.) blinded to genotype.

### Real-time quantitative RT-PCR (Q-RT-PCR)

Total RNA was isolated from either enriched glomerular preparations or podocyte cultures, and the reverse transcription reaction was performed as described^65^. Real-time quantitative PCR was performed using CFX96 PCR machine (BioRad Laboratories, Inc.) and the universal SYBR Green PCR master Mix Kit (Perkin-Elmer Applied Biosystems Division, Wellesley, MA, USA) as previously described^65^. The amplification signals were normalized to the endogenous cyclophilin A mRNA level. The followings sequences were used for the mouse primers: MYH9 – ctaagagcctcgccaagc and gtcttctccagctcctgtc; CTGF – ttggcccagacccaactatg and caggaggcgttgtcattggt; SMAD7 – tgctgtgccttcctccgctg and gccaccacgcaccagtgtga; ANGPTL1 – gccaccacctgatctggcaact and cccactgaccgaatgcccagc; HIF1A - acagcagccagacgatcatgc and actggtcagctgtggtaatccact; MYL9 - catccatgaggaccacctccg and ctggggtggcctagtcgtc; cyclophilin A – ggccgatgacgagccc and tgtctttggaactttgtctgcaa; TESK1 tggacattgcacaagg and cagtccgaagtcacccacaa. The followings sequences were used for the human primers: TESK1 – ccctggacattgcccgagg and cagcccgaagtcacccacga.

### Attachment assays

Attachment assays were performed using a procedure modified from Tsuchida *et al*.^66^. Podocytes were plated on collagen coated 24 well tissue culture clusters coated overnight with 50 µg/ml fibronectin Santa Cruz Biotechnology (product number sc-29011) or a 1% collagen solution from Sigma-Aldrich, Inc. (product number C8919). Non-specific binding was blocked with a 1% albumin solution in PBS. Podocytes were then resuspeded at a concentration of 2.5 × 10^5^ cells/ml in serum free RPMI 1640 at 37 °C in the presence or absence of 100 µM of the β3 integrin small molecule inhibitor cyclo-RDGfV peptide^35^ and incubated for 30 minutes at 37 °C with gentle rocking. The podocyte suspension (200 µl) was then plated in each well and incubated at 37 °C for 1 hour. Cells were washed 3 times with 1 ml of RPMI 1640 at 37 °C, and then cells were harvested and counted using a hemocytometer. Data was expressed as a percentage of the percent input of the cells bound.

### Immunoblotting

Immunoblotting and densitometric analyses were performed using methods adapted from previous studies^60^ with the modification that immunoblotting was performed with antibodies diluted in 5% bovine serum albumin. The following antibodies were obtained from Cell Signaling Technology (Beverly, MA): 1. Phospho-MYPT1 (product number 4563), total MYPT1 (product number 2634), Phospho-CFL1 (product number 3313), total CFL1 (product number 5175), acetyl-α-tubulin (product number 3671), and TESK1 (product number 46552). Additional antibodies were obtained from the following sources and included: 1. Detyrosinated α-tubulin from Millipore (product number AB3201), 2. TESK1 from Thermo-Fisher Scientific (product number PA5-39143), and 3. α-tubulin from Santa Cruz Biotechnology (product number sc-5286). For individual immunoblots, data was normalized to the controls to compensate for exposure differences between studies.

### Rho A pull-down assays

Rho A pull-down assays were performed using a kit from Cytoskeleton, Inc. (Denver, CO) using the protocol described by the manufacturer.

### Immunofluorescence studies

Human kidney sections were obtained from Origene (Rockville, MD). The primary TESK1 antibody was obtained from Thermo-Fisher (Waltham, MA) and the primary synaptopodin antibody was obtained from Progen (Heidelberg, German). The following cross-adsorbed secondary antibodies were obtained from Thermo-Fisher: 1. A goat anti-mouse rhodamine red-x antibody, and 2. A donkey anti-rabbit Alexa Fluor 488 antibody (product number A21206). For the studies, kidney sections were fixed in 1%paraformaldehyde in phosphate buttered saline (PBS) for 5 minutes, washed and then permeabilized with 0.1% Triton X-100 in PBS. Sections were incubated with the primary antibodies overnight in blocking solution, washed and then incubated with the secondary antibodies for 1 hour. After washing, cover slides were applied using Shur/Mount (Triangle Biomedical Sciences, Durham, NC) containing DAPI (4′,6-diamidino-2-phenylindole). Slides were examined using a Nikon Eclipse TE2000-S fluorescent microscope, and digital images obtained with NIS Elements-F imaging software (version 3.2).

### Phalloidin staining

Podocytes were plated on collagen coated 6 well tissue culture clusters and then differentiated as previously described^64^. Expression of polymerized actin was visualized by phalloidin staining using Alexa Fluor 568 phalloidin from Molecular Probes (Eugene, OR), (product number A12380). For the experiments, podocytes were incubated in the indicated culture conditions for 24 hours. Cells were then fixed in 2% paraformaldehyde for 10 minutes and the excess aldehyde quenched with 0.1 M glycine in Dulbecco’s phosphate buffered saline (D-PBS). Cell were permeabilized with 1% Triton-X in D-PBS for 2 minutes and then incubated with Alexa Fluor 568 phalloidin (1:100) in Alexa Fluor 568 phalloidin for 30 minutes. After washing, cover slips were applied using adhesive containing DAPI (4′,6-diamidino-2-phenylindole) and slides examined using a Nikon Eclipse TE2000-S fluorescent microscope. Digital images were obtained with NIS Elements-F imaging software (version 3.2).

### Quantitation of red pixel intensity

For the studies, cells images were digitally obtained with NIS Elements-F imaging software (version 3.2) using an identical exposure duration for all images, unless otherwise noted. Mean red pixel intensity was then measured using Adobe Photoshop CS6 Extended software (Adobe Systems, Inc.). Quantitation of mean red pixel intensity in the images was then performed in an unbiased fashion using Adobe Photoshop CS6 Extended software (Adobe Systems, Inc.) by selecting “window > histogram” and the on the colors drop-down menu in the histogram window “red”.

### KO of TESK1 in murine immortalized podocytes

Podocytes were plated on collagen coated 6 well tissue culture clusters and then differentiated as previously described^64^. For the experiments, we knocked out TESK1 using CRISPR [(Clustered regularly interspaced short palindromic repeats)/CAS9 (CRISPR associated protein 9)] technology^41^ and constructs designed by Origene (Rockville, MD). For the experiments, podocytes were co-transfected with a guide RNA/CAS9 vector and a donor vector containing homologous arms and a functional cassette expressing both green fluorescent protein (GFP) and a puromycin resistance sequence. The donor cassette utilizes a left homologous arm sequence immediately upstream of the TESK1 ATG start site. As a result, GFP expression is driven by the endogenous TESK1 promoter (the phosphoglycerate kinase or PGK promoter drives expression of the puromycin resistance sequence). Following transfection, cells were selected using 8 µg/ml puromycin and individual clones identified by the limiting dilution method. Clones lacking expression of TESK1 mRNA by Q-RT-PCR and expressing GFP were selected for study.

### Statistical analysis

Data are presented as the mean ± standard error of the mean (SEM). For comparison of continuous variables between two groups, statistical significance was assessed by a t-test using the Prism computer program (GraphPad Software, Inc.). For comparisons between more than two groups, statistical analysis was performed using a one way analysis of variance (ANOVA) followed by a Bonferonni multiple comparisons post test.

### Data availability statement

All data generated or analyzed during this study are included in this published article and its Supplementary Information files.

## Electronic supplementary material

Supplementary Information
