# Supplementary material for: Regulation of cofilin phosphorylation in glomerular podocytes by testis specific kinase 1 (TESK1)
Source: Sci Rep. 2018 Aug 16;8:12286. doi: 10.1038/s41598-018-30115-3 (PMC6095849; doi:10.1038/s41598-018-30115-3)
Supplement: Supplementary file 1 — Supplementary Information [file 41598_2018_30115_MOESM1_ESM.pdf]

**Regulation or actin cytoskeletal remodeling in glomerular podocytes by testis specific kinase 1 (TESK1)**


Liming Wang[1], Anne F. Buckley[2], Robert F. Spurney[1]

[1]Division of Nephrology, Department of Medicine, Duke University and Durham VA Medical Centers, Durham, NC, 27710

[2]Department of Pathology, Duke University Medical Center, Durham, NC 27710

```
                  1570      1580      1590      1600      1610      1620
Ms TESK1* CTGCTCAGCTCCCCAGAACCCCTCTCCTGGCCAGTCAGGCTCCACCTAGCCCTGGACATT
                                                           ::::::::::: 
RT-PCR    -------------------------------------------------CCCTGGACATT
                                                                    10

                  1630      1640      1650      1660      1670      1680
Ms TESK1* GCACAAGGCCTACGGTACCTACACGCCAAAGGTGTGTTTCACCGAGACCTCACATCCAAG
          ::::::::::::::::::::::::::::::::::::::::::::::::::::::::::::
RT-PCR    GCACAAGGCCTACGGTACCTACACGCCAAAGGTGTGTTTCACCGAGACCTCACATCCAAG
                  20        30        40        50        60        70

                  1690      1700      1710      1720      1730      1740
Ms TESK1* AACTGTCTGGTCCGAAGGGAAGACCGAGGCTTCACAGCTGTTGTGGGTGACTTCGGACTG
          ::::::::::::::::::::::::::::::::::::::::::::::::::::::::::::
RT-PCR    AACTGTCTGGTCCGAAGGGAAGACCGAGGCTTCACAGCTGTTGTGGGTGACTTCGGACTG
                  80        90       100       110       120       130
```

***NCBI Reference Sequence: NM_011571.3**

Figure S1. Sequence of the murine RT-PCR product. RT-PCR was performed using total cellular RNA from mouse podocytes and intron spanning primers as detailed in the Methods Section. Shown is the alignment of the RT-PCR product with mouse (Ms) TESK1 mRNA sequence (NCBI Reference Sequence NM_011571.3)

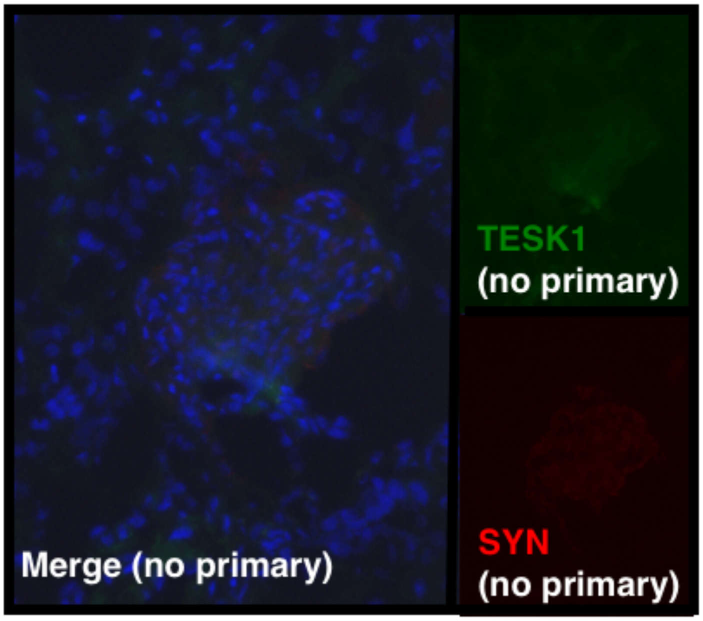


Figure S2. Negative control for immunofluorescent studies. Nuclei in frozen human kidney sections were counterstained with DAPI (blue) in the absence of the monoclonal antibodies to the podocyte marker synaptopodin (SYN)(red) and the monoclonal antibody to TESK1. Nuclei were counterstained with DAPI (blue). TESK1 and SYN staining were not detected in the absence of the primary antibodies.

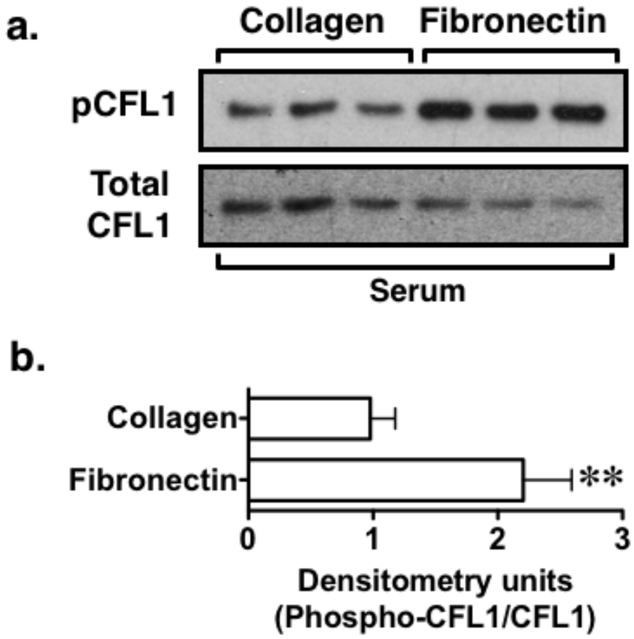


Figure S3. (a-b) Plating podocytes on fibronectin in the present of serum significantly increased pCFL1 levels compared to podocytes plated on collagen. **P<0.01 vs collagen

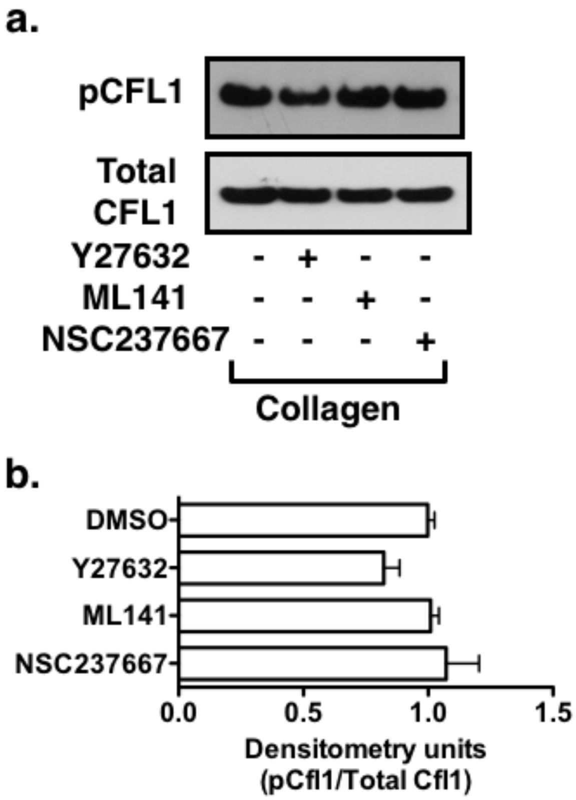


Figure S4. (a-b) Fibronectin induced CFL1 phosphorylation was not significantly inhibited by the ROK inhibitor Y27632, the Cdc42 inhibitor ML141or the Rac1 inhibitor NSC 237667.

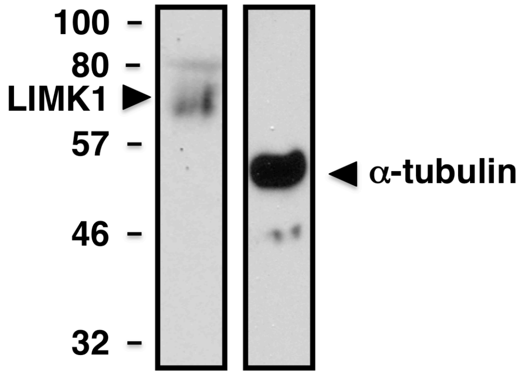


Figure S5. LIMK1 was expressed in cultured mouse podocytes.

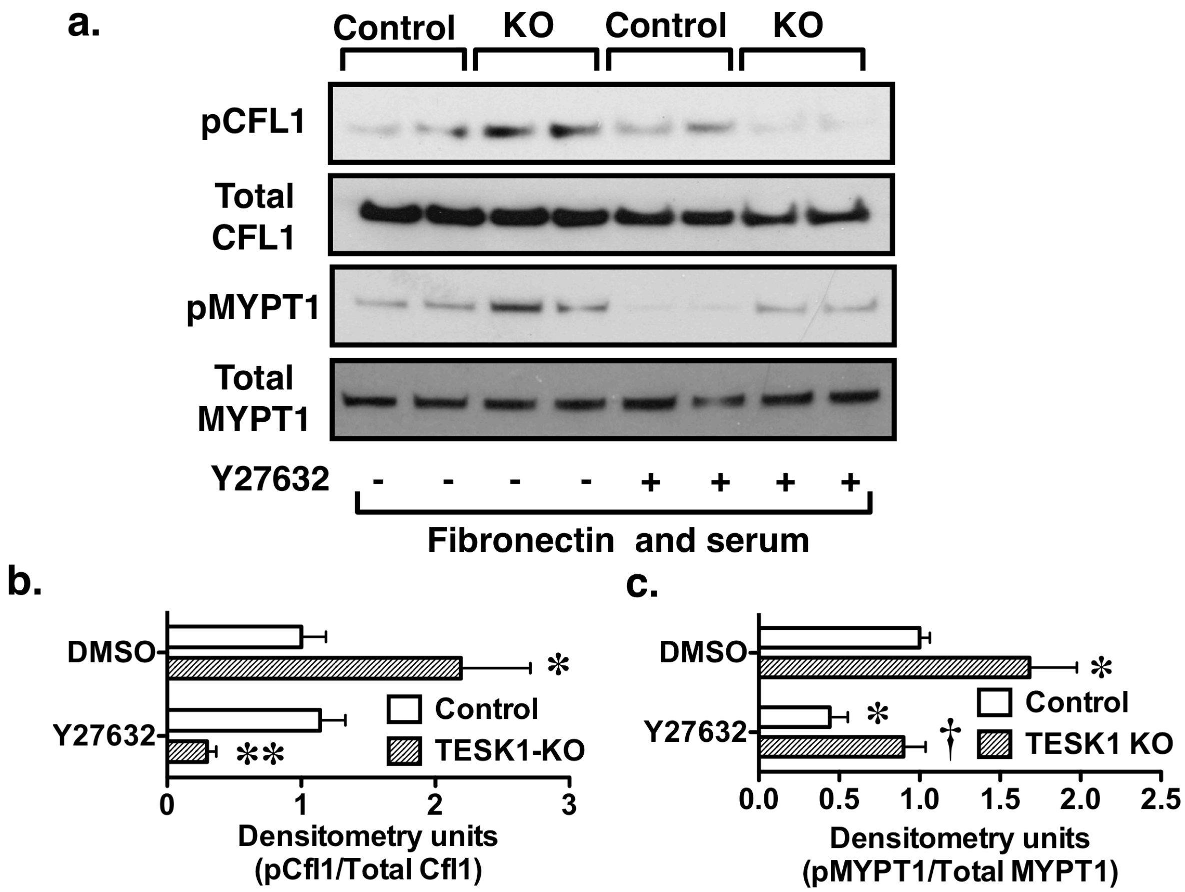


Figure S6. (a-c) KO of TESK1 enhanced CFL1 and pMYPT1 phosphorylation in serum-treated podocytes. TESK1 KO increased pCFL1 levels in mouse podocyte plated on fibronectin in the presence of serum. Treatment with Y27632 (10 µM) had little effect on pCFL1 levels in control podocytes despite effective ROK inhibition (decreased pMYPT1). In contrast, combined ROK inhibition with Y27632 (10µM) and TESK1 KO inhibited CFL1 phosphorylation, consistent with a role for both ROK and TESK1 in promoting CFL1 phosphorylation. Results of 4 independent experiments. *P<0.05 vs control podocytes treated with DMSO, **P<0.01 vs TESK1 KO podocytes DMSO, †P<0.05 vs KO DMSO
